# PReS-FINAL-2162: Development of a risk model to predict methotrexate intolerance in juvenile idiopathic arthritis

**DOI:** 10.1186/1546-0096-11-S2-P174

**Published:** 2013-12-05

**Authors:** P Van Dijkhuizen, M Bulatovic-Calasan, S Pluijm, M De Rotte, B Vastert, S Kamphuis, R De Jonge, N Wulffraat

**Affiliations:** 1 paediatric immunology, UMC Utrecht, Utrecht, Netherlands; 2 Paediatric haemato-oncology, Rotterdam, Netherlands; 3 Clinical Chemistry, Rotterdam, Netherlands; 4 paediatric immunology, Erasmus University Medical Centre, Rotterdam, Netherlands

## Introduction

Methotrexate (MTX) is a cornerstone and safe disease-modifying anti-rheumatic drug (DMARD) in the treatment of juvenile idiopathic arthritis (JIA). Despite its safety, MTX intolerance occurs frequently, leading to non-compliance, thus hampering efficacy and potentially leading to early discontinuation.

## Objectives

The aim of this study was to construct a risk model to predict MTX intolerance.

## Methods

In a large, prospective JIA cohort, potential predictors (clinical variables and single nucleotide polymorphisms) were determined at the time of MTX start. The Methotrexate Intolerance Severity Score was employed to measure MTX intolerance in the first year of treatment. MTX intolerance was most prevalent at 6 or 12 months after MTX start. This was defined as the outcome for the prediction model. The model was developed in 152 patients using multivariate logistic regression analysis. It was subsequently internally validated using bootstrapping.

## Results

The prediction model included the following predictors: JIA subtype, antinuclear antibody, parent/patient assessment of pain, Juvenile Arthritis Disease Activity Score-27, thrombocytes, alanine aminotransferase and creatinine. The model classified 77.5% of patients correctly, changing to 66.7% after internal validation by bootstrapping. The prediction model was transformed into a risk score (range 0-17). At an optimal cut-off of ≥6, sensitivity was 82.0%, specificity 56.1%, positive predictive value was 58.7% and negative predictive value 80.4%.

## Conclusion

The prediction model combined routine clinical variables and showed good predictive power to detect MTX intolerance. This easy-to-use tool could assist clinicians in identifying patients at risk to develop MTX intolerance. Consequently, clinicians can monitor them closely and intervene timely before MTX intolerance hampers MTX efficacy.

## Disclosure of interest

None declared.

